# A Holistic Approach to Model the Kinetics of Photocatalytic Reactions

**DOI:** 10.3389/fchem.2019.00128

**Published:** 2019-03-14

**Authors:** Jonathan Z. Bloh

**Affiliations:** DECHEMA-Forschungsinstitut, Frankfurt am Main, Germany

**Keywords:** photocatalysis, kinetic analysis, high light intensity, temperature effects, heterogeneous photocatalysis, molecular photocatalysis, photoredox catalysis

## Abstract

Understanding and modeling kinetics is an essential part of the optimization and implementation of chemical reactions. In the case of photocatalytic reactions this is mostly done one-dimensionally, i.e., only considering the effect of one parameter at the same time. However, as discussed in this study, many of the relevant reaction parameters have mutual interdependencies that call for a holistic multi-dimensional approach to accurately model and understand their influence. Such an approach is described herein, and all the relevant equations given so that researchers can readily implement it to analyze and model their reactions.

## 1. Introduction

The importance of photocatalysis in fundamental and applied science has expanded tremendously over the last decades (Schneider et al., 2014; Augugliaro et al., 2015; Balzani et al., 2015; Romero and Nicewicz, 2016). Next to applications of heterogeneous photocatalysis in the removal of air pollutants (Ballari and Brouwers, 2013; Patzsch et al., 2018) and waste-water treatment (Alfano et al., 2000; Malato et al., 2009), a variety of applications in the field of organic synthesis (Friedmann et al., 2016; Bloh and Marschall, 2017) have emerged, particularly using molecular photoredox catalysts (Zeitler, 2009; Romero and Nicewicz, 2016).

In the latter field, this interest is in part due to the need of more efficient, sustainable and eco-friendly reactions as photons are essentially traceless reagents, but also partly due to the emergence of LEDs as very affordable and efficient high-power light sources which have made it very easy to perform photocatalytic reactions with appreciable rates. However, if these reactions are to be implemented in industrially relevant processes, they need to be both efficient and productive, i.e., and have both high quantum yield and reaction rates at the same time. This requires a precise knowledge about kinetics and the influence of all relevant reaction parameters. Yet, relatively little is known about the behavior of these reactions at the very high photon fluxes required to reach molar conversions within hours. Generally, at least for heterogeneous reactions, the observation is that at some point the response of the reaction rate to the light intensity becomes non-linear and yields increasingly diminishing returns (Dillert et al., 2013; Dilla et al., 2017; Deng, 2018).

Kinetic modeling and analysis of heterogeneous photocatalysis is mostly based on relatively simple Langmuir-Hinshelwood type kinetics, with linear or mixed linear and square root dependence on the light intensity (Mills et al., 2015; Camera-Roda et al., 2016). The major problem with this approach is that using this rate law, with an average light intensity, is only a valid approach if the reaction rate scales linearly with the light intensity at every point in the reaction vessel. Considering the distribution of the light intensity and absorption inside the reactor and calculating local reaction rates to integrate into a global average, is only done in a select few and very specific cases (Camera-Roda et al., 2016; Grčić and Li Puma, 2017).

In photoredox catalysis, kinetic analysis is almost exclusively based on fluorescence methods such as Stern-Volmer analysis (Arias-Rotondo and McCusker, 2016; Pitre et al., 2016).

As shown recently, most reaction parameters show a mutual interdependence on each other and therefore cannot be properly studied in one-dimensional approaches, where just one parameter is varied (Burek et al., 2019). Instead, a holistic approach is needed to take the light intensity and distribution, catalyst and substrate concentration and the temperature into account at the same time. This contribution describes this approach in detail.

## 2. Heterogeneous Photocatalytic Reactions

**Figure 1:**
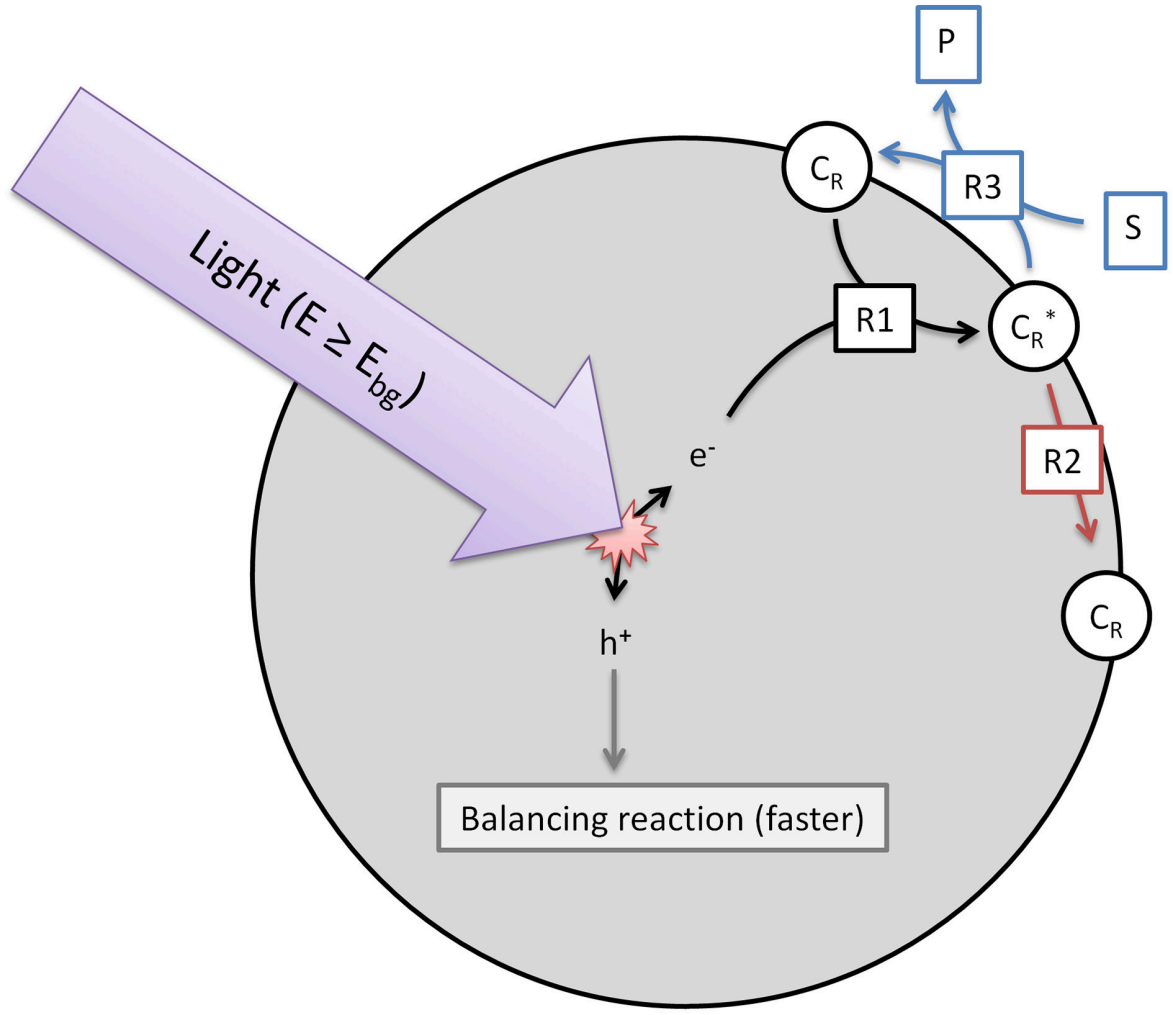
Illustration of the three elementary reactions which are the basis of the kinetic model. Shown are the generation of reactive surface sites (*R*1), the recombination (*R*2) and the charge transfer to the target substrate (*R*3).

The model used herein to describe heterogeneous photocatalytic reactions is based on three elementary steps (*R*1-3), which are illustrated in Figure 1. While in the overall reactions, reduction and oxidation always have to take place, we only consider one of those half-reactions, whichever one is slower and rate-determining. The other half-reaction is consequently assumed to be much faster and has no effect on the overall observed reaction rate. It should also be considered that contrary to molecular systems, reduction and oxidation neither have to take place in a set sequence, nor necessarily at the same time, as the photocatalyst particle has a certain capacity to store excess charges over short time-frames (Mohamed et al., 2011).

The first reaction step (*R*1, Equation 1) is the generation of reactive surface sites ($c_{R}^{*}$) which are essentially charge carriers (electrons or holes, depending on whichever reaction is rate-limiting) trapped at surface sites. The rate of this reaction is dependent on the local volumetric rate of photon absorption (LVRPA, $L_{p}^{a}$), the quantum yield (ϕ) and is normalized to the fraction of available surface trap sites that are not already filled. This is based on the assumption that a trap already filled cannot take up additional charges.

Many kinetic models assume that the reaction rate is dependent on the concentration of conduction band electrons (or valence band holes). However, this approach is flawed since the electrons and the substrate molecules are present in separate phases and can therefore not freely interact with each other. Since the reaction happens *via* the surface, only electrons present (or trapped) at the surface matter for the reaction rate. We simply consider the whole process of photon absorption, electron/hole pair generation and migration and trapping at surface sites as one process. Consequently, the respective quantum yield (ϕ) already comprises all loss processes along this pathway, e.g., bulk recombination. The efficiency of this process should theoretically be a property of the photocatalyst and be independent of the reaction studied. Additionally, the normalization to the total number of available surface trap sites is necessary, otherwise, given a high enough light intensity the model could theoretically create an infinite number of reactive active sites, which will never be the case for a limited number of catalyst particles.

(1) $$\begin{array}{l} R1\left(c_{R}\to c_{R}^{*}\right):\phi \cdot L_{p}^{a}\cdot \frac{c_{R}}{c_{R,0}} \end{array}$$

These reactive surface sites can relax back to the ground state by recombining with their respective charge-carrier counterpart (*R*2, Equation 2). Note that this represents only the (relatively slow) decay of surface traps, as bulk recombination is already accounted for in *R*1. We model this as a simple first-order reaction, dependent on the recombination rate (*k*_*r*_) and the density of trapped charges, given here by the fraction of filled surface traps to total surface traps.

Often, the recombination rate is modeled as a second-order reaction with respect to the concentration of electrons or holes (Zhang et al., 2012). This is a problematic approach, as the concentration (in electrons per reaction volume unit) of charges is not an appropriate measure since the electrons cannot freely move inside the reaction medium, only inside their photocatalyst (nano)particle. If for instance, in a given volume element there are 10 photocatalyst particles each containing a single charge, the recombination rate should be different than if it is just one particle containing 10 charges. It is for this reason that we chose to model the recombination rate as a function of density of trapped charges (in the photocatalyst particles) rather than a concentration of charge carriers. It should be noted however, that for an invariant catalyst concentration this is still proportional to the concentration of charges.

There is considerable disagreement in the literature about whether the decay of charge carriers follows first- or second-order kinetics (Zhang et al., 2012). In this case, while *R*2 models this as a first-order decay, the total rate of recombination (factoring in the contribution of bulk recombination from *R*1) is actually predominantly second-order with respect to the light intensity, *cf*. Burek et al. (2019), SI.

(2) $$\begin{array}{l} R2\left(c_{R}^{*}\to c_{R}\right):k_{r}\cdot \frac{c_{R}^{*}}{c_{R,0}} \end{array}$$

Finally, the charge transfer to the target substrate can be considered (*R*3, Equation 3). This is dependent on the concentration of reactive surface sites, the surface coverage (θ) of the photocatalyst with the target substrate and a monomolecular kinetic constant (*k*). The surface coverage can simply be modeled as a function of substrate concentration ([*S*]) and adsorption constant (*K*_*ads*_) using a Langmuir isotherm, Equation (4).

(3) $$\begin{array}{l} R3\left(c_{R}^{*}+S\to c_{R}+P\right):k\cdot \theta \cdot c_{R}^{*} \end{array}$$

(4) $$\begin{array}{l} \theta =\frac{K_{ads}\cdot \left[S\right]}{1+K_{ads}\cdot \left[S\right]} \end{array}$$

Under the assumption that these processes (*R*1-3) happen on a timescale much faster than macroscopic mixing and changes in the substrate concentration, a pseudo-steady-state approach ($c_{R},c_{R}^{*}=const.$, *R*1 = *R*2 + *R*3) yields an explicit equation for the concentration of reactive surface sites, Equation (5), and the target (observed) reaction rate (*r* = *R*3), Equation (6).

(5) $$\begin{array}{l} c_{R}^{*}=\frac{\phi \cdot L_{p}^{a}\cdot c_{R,0}}{\phi \cdot L_{p}^{a}+k_{r}+k\cdot \theta \cdot c_{R,0}} \end{array}$$

(6) $$\begin{array}{l} r=R3=\frac{\phi \cdot L_{p}^{a}\cdot c_{R,0}\cdot k\cdot \theta }{\phi \cdot L_{p}^{a}+k_{r}+k\cdot \theta \cdot c_{R,0}} \end{array}$$

Since the concentration of reactive sites is difficult to determine and typically unknown, it is more practical to normalize the rate constant to the catalyst mass (*c*_0_), Equation (7), which leads to Equation (8), which represents the general case for calculating the *local* reaction rate. It is important to understand that this equation cannot be directly used to calculate observed *average* reaction rates unless certain criteria are met (*vide infra*), since the local reaction rate dramatically varies throughout the reaction medium.

(7) $$\begin{array}{l} k^{*}=\frac{k\cdot c_{R,0}}{c_{0}} \end{array}$$

(8) $$\begin{array}{l} r=\frac{\phi \cdot L_{p}^{a}\cdot k^{*}\cdot \theta \cdot c_{0}}{\phi \cdot L_{p}^{a}+k_{r}+k^{*}\cdot \theta \cdot c_{0}} \end{array}$$

### 2.1. Effect of Catalyst Concentration and Light Intensity

**Figure 2:**
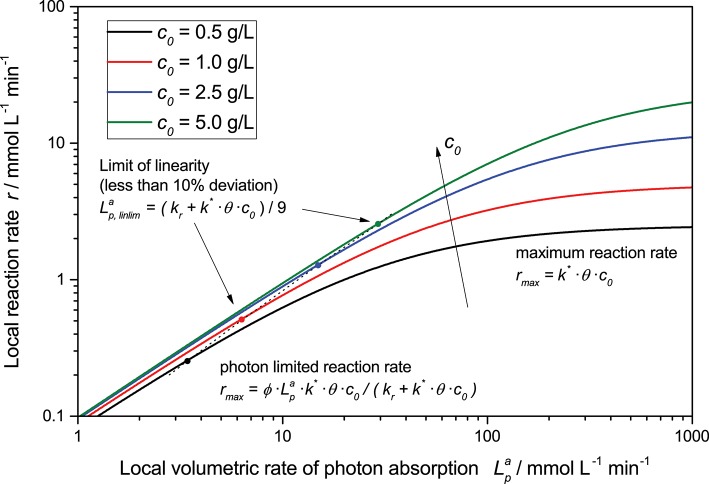
Exemplary dependency of the local reaction rate (Equation 8) on the light intensity for different catalyst concentrations in a double logarithmic plot. Parameters used: *ϕ* = 0.1, *k_r_* = 500 μmol L^−1^ min^−1^, k^*^ = 5.000 μmol g^−1^ min^−1^, *θ* = 1.

This general rate law (Equation 8) can be simplified if one of the limiting cases implies that the light intensity is either very high or very low in relation to the reaction's kinetic limit, Equations (9) and (10), these limiting cases can also be seen in Figure 2. At low light intensity, the reaction is purely governed by the flux of absorbed photons, in this regime, the observed overall photonic efficiency is constant. Parameters affecting the rate of electron transfer from the photocatalyst to the substrate have only negligible effect here. If on the contrary, the local light intensity is very high, the reaction is completely limited by the intrinsic kinetics of the reaction. In this case, the photocatalyst effectively behaves like an ordinary heterogeneous catalyst, since all of the reactive sites are permanently active due to the high photon flux.

(9) $$\begin{array}{l} \left(\phi \cdot L_{p}^{a}\right)\gg \left(k_{r}+k^{*}\cdot \theta \cdot c_{0}\right):r=k^{*}\cdot \theta \cdot c_{0} \end{array}$$

(10) $$\begin{array}{l} \left(\phi \cdot L_{p}^{a}\right)\ll \left(k_{r}+k^{*}\cdot \theta \cdot c_{0}\right):r=\phi \cdot L_{p}^{a}\cdot \frac{k^{*}\cdot \theta \cdot c_{0}}{k_{r}+k^{*}\cdot \theta \cdot c_{0}} \end{array}$$

It is very important to understand that only if the former case of low light intensity and constant photonic efficiency is fulfilled in the whole reaction medium (i.e., even at the point of highest absorbed photon flux), the local reaction rate equals the average (observed) reaction rate 〈*r*〉 when using the average volumetric rate of photon absorption (AVRPA, $\langle L_{p}^{a}\rangle$) instead of the LVRPA, Equation (11). However, particularly for reactions with bad kinetics this will often not be the case even for moderate light intensities (*vide infra*).

(11) $$\begin{array}{l} \left(\phi \cdot L_{p,max}^{a}\right)\ll \left(k_{r}+k^{*}\cdot \theta \cdot c_{0}\right):\langle r\rangle =\phi \cdot \langle L_{p}^{a}\rangle \cdot \frac{k^{*}\cdot \theta \cdot c_{0}}{k_{r}+k^{*}\cdot \theta \cdot c_{0}} \end{array}$$

**Figure 3:**
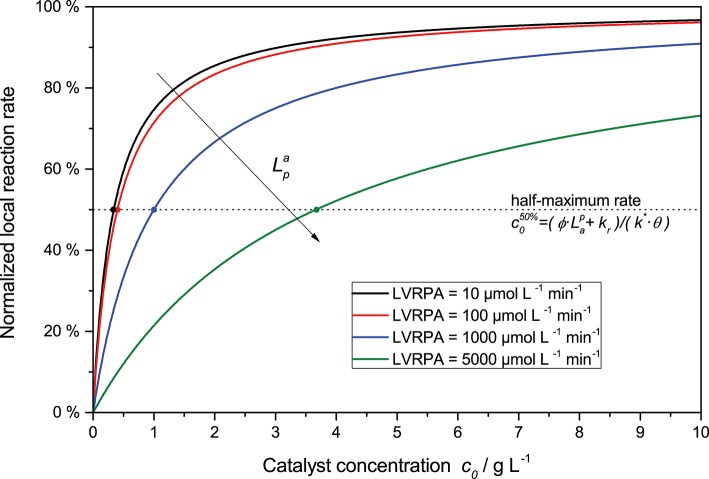
Exemplary dependency of the (normalized) local reaction rate (Equation 8 divided by $L_{p}^{a}$) in dependence of the catalyst concentration for various LVRPA. Parameters used: *ϕ* = 1, *k_r_* = 500 μmol L^−1^ min^−1^, k^*^ = 1.500 μmol g^−1^ min^−1^, *θ* = 1.

Furthermore, the rate equation (Equation 8) shows a saturation-curve behavior with respect to both the light intensity and the catalyst concentration. Interestingly, the value of one of those parameters needed to achieve saturation increases with higher values of the other. This is illustrated in Figure 3, where it is obvious that the catalyst concentration needed for saturation increases linearly with the light intensity. Likewise, if the reaction rate is considered as a function of light intensity, one can see that the higher the catalyst concentrations is, the longer the linear regime and the more light is needed to be fully saturated with photons, *cf*. Figure 2. This inter-dependency of catalyst concentration and light intensity was recently observed by us for the first time in the case of photocatalytic hydrogen peroxide formation by reduction of molecular oxygen (Burek et al., 2019). Since typically, the two parameters are not studied in depth at the same time, this effect has been largely invisible up to now. The conclusion here is that the higher the employed light intensity is, the higher should also be the catalyst concentration in order to keep the same efficiency. Since there are obvious limits to this in terms of solubility/dispersibility of the photocatalyst in the reaction medium, other measures should also be taken at very high light intensities (*vide infra*).

### 2.2. Effect of Substrate Concentration

The local reaction rate equation (Equation 8) can be rewritten into a pseudo-Langmuir-Hinshelwood form, Equation (12). Consequently, if the substrate concentration is the only parameter varied, the behavior might look like classical Langmuir-Hinshelwood and can be modeled and analyzed this way. This approach, however, might lead to false conclusions, as the respective parameters *k*′ and $K_{ads}^{\prime }$ do not resemble their classical physical meaning and are, in fact, influenced by a number of other parameters, *cf*. Equation (13).

(12) $$\begin{array}{l} r=\frac{k^{\prime }\cdot K_{ads}^{\prime }\cdot \left[S\right]}{1+K_{ads}^{\prime }\cdot \left[S\right]} \end{array}$$

(13) $$\begin{array}{l} k^{\prime }=\frac{\phi \cdot L_{p}^{a}\cdot k^{*}\cdot c_{0}}{\phi \cdot L_{p}^{a}+k_{r}+k^{*}\cdot c_{0}};K_{ads}^{\prime }=K_{ads}\cdot \left(1+\frac{k^{*}\cdot c_{0}}{\phi \cdot L_{p}^{a}+k_{r}}\right) \end{array}$$

The fact these pseudo-Langmuir-Hinshelwood parameters are dependent on the light intensity for instance, has already been suggested and experimentally observed by other authors (Ollis, 2005; Dillert et al., 2012; Mills et al., 2015; Camera-Roda et al., 2016).

**Figure 4:**
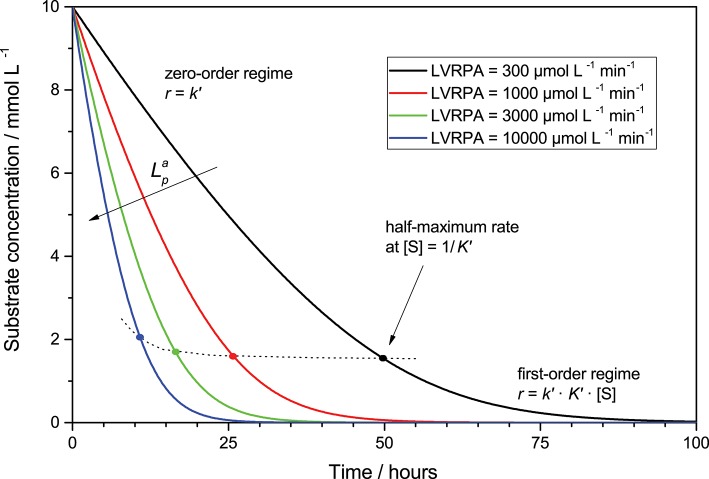
Exemplary course of the substrate concentration (starting from 10 mmol L^−1^) over time in a given volume element for different LVRPA according to Equation (12). Parameters used: *ϕ* = 0.026, *c*_0_ = 2.5 g L^−1^, *k_r_* = 653 μmol L^−1^ min^−1^, k^*^ = 1358 μmol g^−1^ min^−1^, *K_ads_* = 0.1 L mmol^−1^.

Consequently, the observed reaction is rate mixed zero- and first-order at high and low substrate concentration, respectively, *cf*. Figure 4. Interestingly, due to the light intensity dependence of the pseudo-adsorption constant, the inflection points between the zero- and first-order regimes given by the half-maximum rate gradually shifts to higher substrate concentration with higher light intensity. If concentration-time profiles for a given reaction are recorded, they can be modeled and analyzed using Equations (8) or (12), to extract useful information about the respective parameters. Since integration of this rate law does not yield an explicit equation, modeling has to be done numerically using for instance the Euler-Cauchy method. While the possibility to analyze these kinetics using a linearized approach exits, the author advises against that and to use the numerical approach instead, since linearization suffers from error inversion and weighting problems.

### 2.3. Average Reaction Rates

In many cases, rate laws similar to the ones presented above are applied to analyze the concentration-time profiles of photocatalytic reactions. However, this completely ignores the fact that these are *local* reaction rates, depending on the *local* absorbed photon flux, which varies dramatically throughout the reaction medium. As mentioned above, using the average photon flux to calculate the average reaction rate is only valid if the reaction rate is linearly dependent on the light intensity even at the “brightest spot” of the reaction, i.e., where the absorbed photon flux is at its maximum, Equation (11). In a linear light path, by ignoring scattering and calculating the light absorption behavior using the Lambert-Beer law, Equation (14), the LVRPA can be approximated using the negative derivative of the intensity attenuation, Equation (15), with the irradiance (*I*_0_, given in photons, not energy) and the extinction coefficient (ϵ). From this, the maximum locally absorbed light intensity at the very beginning of the light path (*z* = 0) can be calculated using Equation (16).

(14) $$\begin{array}{l} I\left(z\right)=I_{0}\cdot 10^{-\epsilon \cdot c_{0}\cdot z} \end{array}$$

(15) $$\begin{array}{l} L_{p}^{a}\left(z\right)=\frac{-dI\left(z\right)}{dz}=\epsilon \cdot c_{0}\cdot ln\left(10\right)\cdot I_{0}\cdot 10^{-\epsilon \cdot c_{0}\cdot z} \end{array}$$

(16) $$\begin{array}{l} L_{p,max}^{a}=L_{p}^{a}\left(0\right)=\epsilon \cdot c_{0}\cdot ln\left(10\right)\cdot I_{0} \end{array}$$

With this equation it becomes apparent that the maximum locally absorbed photon flux can easily be 2 to 3 orders of magnitude higher than the average. Consequently, only if the condition given by Equation (17) is met, the above mentioned simplification of using Equation (11) is valid. Experimentally this can be checked by studying the reaction rate's response to a varied light intensity. If the response is completely linear across the studied range, the simplified approach is allowed.

(17) $$\begin{array}{l} \left(\phi \cdot \epsilon \cdot c_{0}\cdot ln\left(10\right)\cdot I_{0}\right)\ll \left(k_{r}+k^{*}\cdot \theta \cdot c_{0}\right) \end{array}$$

However, if this not the case, then the local reaction rate needs to be integrated over the whole reaction medium, taking the LVRPA distribution into account to obtain the average observed reaction rate. Particularly for complex light source and reactor geometries, this can be a quite challenging and time-consuming task which involves calculating the LVRPA across the three-dimensional reaction volume with numerical methods. However, under certain conditions this can be vastly simplified: If the light path from the lamp is collimated and is only attenuated along one dimension of the reactor. This is for instance the case when using a tubular reactor that is irradiated from its circular base or top, for instance when using a standard cylindrical beaker and irradiating it from above. In this case, the LVRPA can be approximated to only vary alongside the direction of the beam (*z*) with the other two directions following rotational symmetry. This allows to estimate the LVRPA using Equation (15) and to calculate the average reaction rate by integrating the local reaction rate over the whole reactor volume, Equation (18), yielding Equation (19).

(18) $$\begin{array}{l} \langle r\rangle =\frac{1}{d}\cdot \int_{0}^{d}\frac{\phi \cdot L_{p}^{a}\left(z\right)\cdot k^{*}\cdot \theta \cdot c_{0}}{\phi \cdot L_{p}^{a}\left(z\right)+k_{r}+k^{*}\cdot \theta \cdot c_{0}}dz \end{array}$$

(19) $$\begin{array}{l} \langle r\rangle =k^{*}\cdot \theta \cdot c_{0}+\frac{k^{*}\cdot \theta }{d\cdot \epsilon \cdot ln\left(10\right)}\cdot ln\\ \;\left(\frac{\phi \cdot I_{0}\cdot \epsilon \cdot c_{0}\cdot ln\left(10\right)+k^{*}\cdot \theta \cdot c_{0}+k_{r}}{\phi \cdot I_{0}\cdot \epsilon \cdot c_{0}\cdot ln\left(10\right)+\left(k^{*}\cdot \theta \cdot c_{0}+k_{r}\right)\cdot 10^{\epsilon \cdot c_{0}\cdot d}}\right) \end{array}$$

Unless very optically dilute solutions are used, the condition expressed by Equation (20) is true and a simplified version of the rate equation can be used, Equation (21).

(20) $$\begin{array}{l} 10^{\epsilon \cdot c_{0}\cdot d}\gg \frac{\phi \cdot I_{0}\cdot \epsilon \cdot c_{0}\cdot ln\left(10\right)}{k^{*}\cdot \theta \cdot c_{0}+k_{r}} \end{array}$$

(21) $$\begin{array}{l} \langle r\rangle =\frac{k^{*}\cdot \theta }{d\cdot \epsilon \cdot ln\left(10\right)}\cdot ln\left(\frac{\phi \cdot I_{0}\cdot \epsilon \cdot c_{0}\cdot ln\left(10\right)}{k^{*}\cdot \theta \cdot c_{0}+k_{r}}+1\right) \end{array}$$

Furthermore, by defining the optical density (α) and using the volumetric photon flux density (*q*_*p*_) instead of the irradiance, Equation (22), the sometimes more practical variant Equation (23) is obtained.

(22) $$\begin{array}{l} I_{0}=q_{p}\cdot d;\alpha =\epsilon \cdot d\cdot ln\left(10\right) \end{array}$$

(23) $$\begin{array}{l} \langle r\rangle =\frac{k^{*}\cdot \theta }{\alpha }\cdot ln\left(\frac{\phi \cdot q_{p}\cdot \alpha \cdot c_{0}}{k^{*}\cdot \theta \cdot c_{0}+k_{r}}+1\right) \end{array}$$

**Figure 5:**
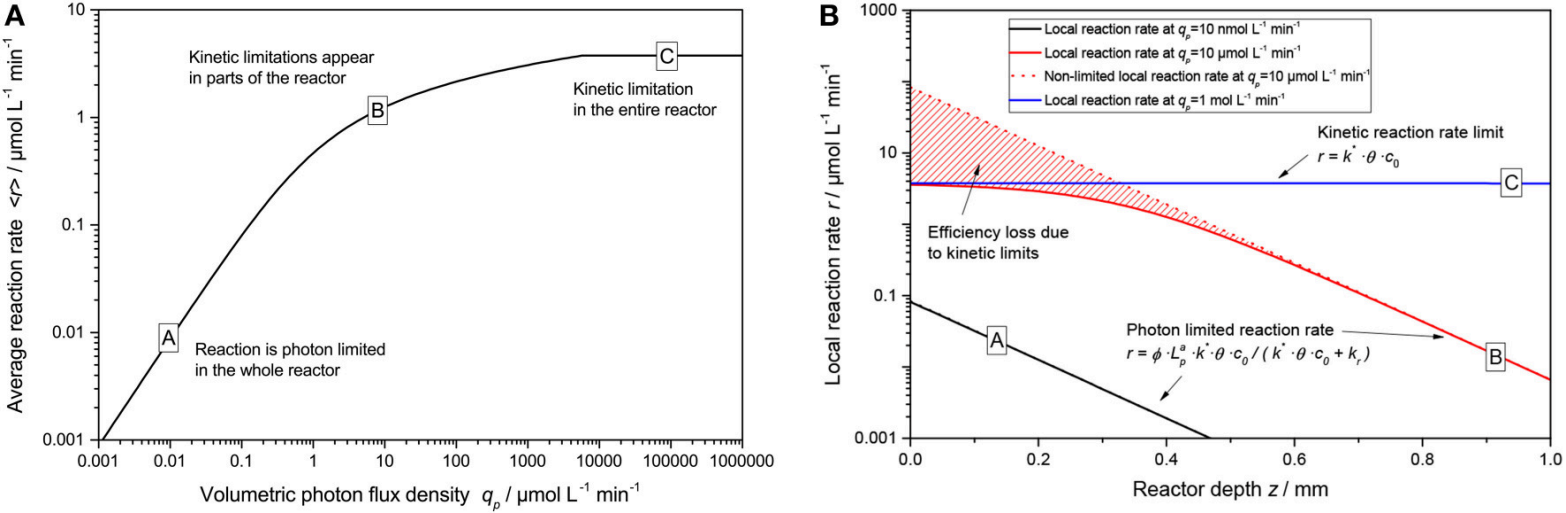
**(A)** Exemplary course of the average reaction rate 〈*r*〉 in dependence of the volumetric photon flux density *q*_*p*_ according to Equation (19). **(B)** Locally resolved reaction rate as a function of reactor depth *z* according to Equation (8) with Equation (15) for the three cases marked A–C in **(A)**. Parameters used: *ϕ* = 1, *c*_0_ = 2.5 g L^−1^, *k_r_* = 500 μmol L^−1^ min^−1^, k^*^ = 1.500 μmol g^−1^ min^−1^, *θ* = 1, *ϵ* = 16.4 L g^−1^ cm^−1^, *d* = 0.1 cm.

An exemplary plot of Equation (19) is shown in Figure 5A. Here, three regimes are apparent: At low light intensity, the reaction scales linearly with the light intensity across the whole reaction medium (A), in this case the simplified rate law given by Equation (11) is applicable. At some point, non-linearities start to appear in some parts of the reactor (the “bright” zone) and the average reaction rate starts to show diminishing returns (B). This corresponds to the non-linear response (approximately following a square root dependence) that was often reported for photocatalytic reactions at higher light intensities. If extremely high light intensities are used, the reaction will be limited at every point in the reactor and the average reaction rate is given by Equation (9) (C). However, the latter case is practically impossible to achieve with conventional light sources unless very small reactors with dilute photocatalyst are used.

**Figure 6:**
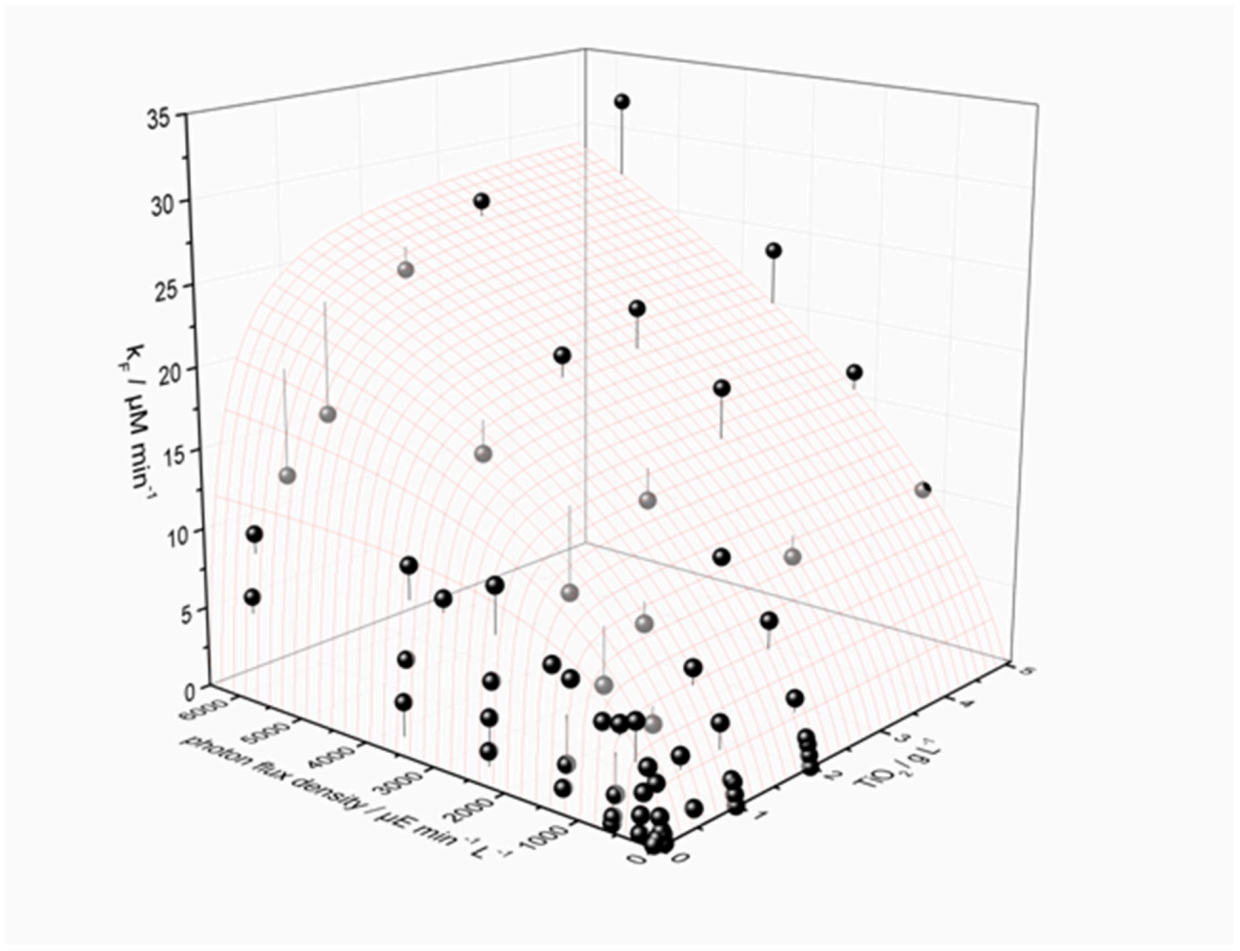
Interdependence of the measured H_2_O_2_ formation rate constant *k*_*F*_ on the amount of catalyst and the photon flux density shown as black and dark gray dots. Also shown is the calculated best fit to the proposed model (Equation 23) as a surface plot; the vertical lines attached to the data points show their respective difference to the calculation. Data points that are black mean they are on or above the surface plot, and dark gray data points signify they are below the surface. Parameters used: *ϕ* = 0.02623, *k_r_* = 653 μmol L^−1^ min^−1^, k^*^ · *θ* = 1358 μmol g^−1^ min^−1^, *ϵ* = 16.4 L g^−1^ cm^−1^, *d* = 4 cm. Reprinted with permission from Burek et al. (2019), ©2018 American Chemical Society.

Recently, we could show on the basis of the photocatalytic reduction of molecular oxygen to hydrogen peroxide, that this approach yields very good results in describing the complex behavior of the reaction rate in dependence of both catalyst concentration and light intensity, *cf*. Figure 6 (Burek et al., 2019).

Figure 5B nicely illustrates the fundamental problem of intensifying photocatalytic reactions. In order to avoid kinetic limitations, the light intensity needs to be so low that even at the beginning of the reactor, no limitations manifest. This in turn means that in the rest of the reactor, the reaction only proceeds with orders of magnitude lower rates. One way to circumvent that is by using very dilute solutions or small dimensions that the light falloff through the reactor is only small (e.g., < 50 %). In that case, the reaction rate would not vary so much in the reactor and the average reaction rate is almost equal to the maximum local reaction rate, vastly increasing catalyst efficiency. However, this would also mean that a significant portion of the light is transmitted though the reactor and not used, dramatically reducing the overall photonic efficiency. Another possible solution is to use delocalized internal illumination so that the light distribution in the medium is more homogeneous (Burek et al., 2017).

### 2.4. Effects of Temperature

Another important parameter of the reaction is the temperature. While this is one of the most important parameters in thermally activated catalysis and typically described via the Arrhenius law, Equation (24), this parameter is only studied in few photocatalytic systems. The rationale behind this seems to be that the reaction is initiated by the massive energy provided by the photons, so room temperature is sufficient to drive the reactions (Herrmann, 1999; Carp et al., 2004; Gaya and Abdullah, 2008; Malato et al., 2009). Yet, there are many publications which clearly show a temperature dependence of the photocatalytic reactions and use the Arrhenius law to calculate apparent activation energies ($E_{A}^{*}$) of 5 to 28 kJ mol-1 (Al-Sayyed et al., 1991; Hirakawa et al., 2004; Soares et al., 2007; Costacurta et al., 2010; Hu et al., 2010). While these are modest values in comparison with typical catalytic processes, they should not be neglected either. For instance, with these values, increasing the reaction temperature from 25 to 80 °C corresponds to an increase of 37 to 481 % in the kinetic constant.

(24) $$\begin{array}{l} k^{*}=A\cdot e^{\frac{-E_{A}}{R\cdot T}} \end{array}$$

**Figure 7:**
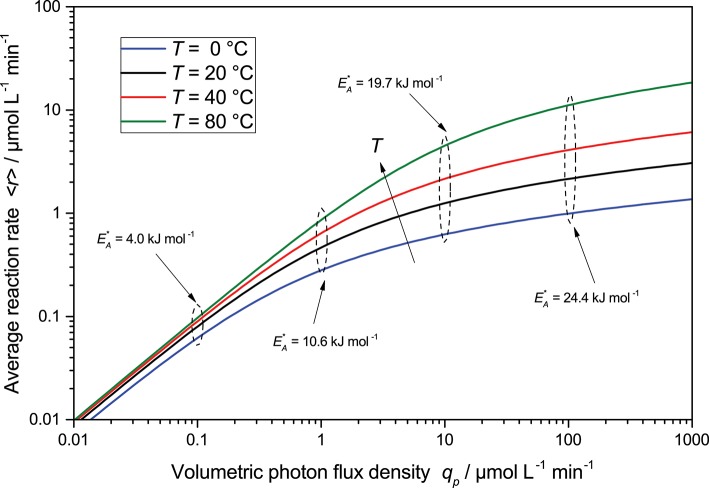
The change in average reaction rate is a function of light intensity for different temperatures calculated according to Equation (23) with Equation (24). Also shown are the apparent activation energies $E_{A}^{*}$ obtained from Arrhenius plots at a given light intensity. Parameters used: *ϕ* = 1, *c*_0_ = 2.5 g L^−1^, *k_r_* = 500 μmol L^−1^ min^−1^, *A* = 3.325 × 10^8^ μmol g^−1^ min^−1^, *E_A_* = 30 kJ mol^−1^, *θ* = 1, *ϵ* = 16.4 L g^−1^ cm^−1^, *d* = 0.1 cm.

The Arrhenius law can easily be integrated into the present kinetic model by modulating the kinetic constant k^*^ by the temperature according to Equation (24). As shown in exemplary Figure 7, the temperature effects are strongly masked at lower light intensities, as improving the kinetics in that regime has negligible impact on the overall reaction, which is limited by the available photons here. However, at high light intensities, the temperature effect becomes much more prominent as it presents a way to overcome kinetic limitation there and to extend the linear regime. Consequently, using higher temperatures is a promising approach to combine high productivity and high quantum yield.

Interestingly, attempts to calculate the apparent activation energies ($E_{A}^{*}$) from the reaction rates obtained at a given light intensity, through Arrhenius plots (they mostly show acceptable fits), yields dramatically differing results with changing light intensity and range from 4.0 to 24.4 kJ mol^−1^. The true activation energy (30 kJ mol^−1^) is only revealed at very high light intensity, when the reaction is entirely kinetically limited. This would explain why authors in the past have reported vastly different apparent activation energies for the same reactions. Consequently, temperature effects of photocatalytic reaction cannot be properly studied at low reaction rates and will only yield misleading results.

Coincidentally, as at very high light intensities the energy input into the solution by the photons will be quite significant, achieving those higher temperatures will often be possible without additional heating. Researchers also need make sure to accurately control and measure the temperature in their reaction media, as to not mistake high light intensity effects for temperature effects that occur through unintentional radiative heating.

As pointed out by some authors, the solubility and adsorption of reactants is also strongly dependent on the temperature and this can significantly contribute to the observed overall temperature effects (Herrmann, 2005; Malato et al., 2009).

### 2.5. Limitations

It should be noted that while the above-mentioned approach has the advantage that it is very easy to implement given the low number of parameters and explicit equations, it also suffers from several drawbacks. The first is that here, a steady-state is assumed and therefore, dynamic processes are not taken into account. For instance, mass transport, adsorption and desorption rates are supposed to be much faster than the reaction are therefore have no effect, only their equilibrium state is modeled. This will obviously not be true in all cases, particularly for very intensified reactions and reagents with sluggish mobility. In those cases, dynamic models have to be used which will unfortunately make complex numerical simulation mandatory.

Furthermore, in the model we consider all photocatalytic reactions as one-electron transfers. If true concerted multi-electron transfers, not consecutive one-electron transfer events, drive the reaction (which typically requires a co-catalyst) this approach likely has to be modified accordingly.

Another potential problem is that scattering is completely neglected in the model (at least in the integrated form). Heterogeneous catalysts suspended in the reaction medium will scatter some of the incident light back out of the reactor, diminishing the actual available photon flux. This will lead to an underestimation of the LVRPA and consequently the quantum yield in the model. However, if the scattered-out light is determined either experimentally or computationally, the incident photon flux can be corrected for it and the model should be accurate with respect to this aspect.

The model also cannot account for changes in the solution properties such as solvent or pH. Since these parameters have very reaction-specific effects, they need to be studied on a case-by-case basis. However, it should be noted that theoretically, changes in the solvent or pH should only affect the rate constant, not the quantum yield or recombination rate.

If different photocatalysts are studied (different materials, surface area, synthesis routes, doping, etc.), this will most likely affect all three primary parameters (rate constant, quantum yield and recombination rate). Since this model requires a substantial amount of data points to deliver fairly accurate parameters, it will be quite time-consuming to compare different catalysts based on the kinetic model. However, doing so would likely provide interesting insight into their properties as changes in the kinetics (rate constant) and photophysical properties (quantum yield, recombination rate) can be separately attributed.

### 2.6. Application of the Model

Despite the limitations stated above, the model presented herein should be readily applicable to most standard photocatalytic reactions. Since most experimental setups feature illumination only from one direction, they are already compatible with the model. Ideally the light source should be collimated, and the beam should hit the reactor at a flat surface, although deviations from that likely only lead to small errors due to higher reflection losses. In almost all cases, Equation (23) can be directly used to model the observed reaction rates. For extremely high light intensities or very low photocatalyst concentrations, it should be checked if Equation (19) yields different values, in that case it has to be used instead.

(23) $$\begin{array}{l} \langle r\rangle =\frac{k^{*}\cdot \theta }{\alpha }\cdot ln\left(\frac{\phi \cdot q_{p}\cdot \alpha \cdot c_{0}}{k^{*}\cdot \theta \cdot c_{0}+k_{r}}+1\right) \end{array}$$

This equation features the typically known parameters catalyst concentration (*c*_0_) and incident light intensity (*I*_0_, measurable for instance with physical probes at the reactor position) or volumetric photon flux density (*q*_*p*_, measurable with chemical actinometry, see Equation (22) about how to interconvert these quantities). The optical density α can also be calculated using Equation (22) and determining the extinction coefficient (ϵ) by a simple transmission measurement. The surface coverage θ can be obtained using Equation (4) which adds the (known) substrate concentration and adsorption constant to the variables. If the substrate concentration is not varied, a constant, e.g., θ = 1, may be used instead but this will scale the rate constant accordingly.

(4) $$\begin{array}{l} \theta =\frac{K_{ads}\cdot \left[S\right]}{1+K_{ads}\cdot \left[S\right]} \end{array}$$

This only leaves the rate constant (k^*^), quantum yield (ϕ) and recombination rate (*k*_*r*_) as unknown variables. These can be obtained by fitting the model to experimental data using non-linear optimization. Since there are three unknown parameters (four if the adsorption constant is unknown as well) the number of data points with varied light intensity and catalyst concentration should be significantly higher than that number to achieve good accuracy for the parameters. The rate equation can also be used to simulate concentration-time-profiles of individual experiments by numerical methods such as the Euler-Cauchy algorithm.

If the effect of changing temperature should be accounted for as well, Equation (24) can be used to replace k^*^ in Equation (23). This adds the known parameter temperature and allows to determine the unknown pre-exponential factor *A* and activation energy *E*_*A*_.

(24) $$\begin{array}{l} k^{*}=A\cdot e^{\frac{-E_{A}}{R\cdot T}} \end{array}$$

Thorough analysis of the model parameters will reveal ways to optimize the reaction with respect to both high reaction rates and photonic efficiency. Typically, this will involve increasing substrate and photocatalyst concentrations to their saturation point as well as the temperature. However, these measures all have physical constraints due to solubility/dispersibility limits and in case of temperature, boiling point of the solvent. So, at a certain point, the reaction cannot be further improved by just tuning the reaction conditions and other measures have to be taken. This can either be improvements of the catalyst itself (which can directly increase the reaction rate kinetics, i.e., k^*^) for instance by using co-catalysts which improve the charge transfer to the substrate. Another way is to improve the reactor used in order to reduce inhomogeneities in the light distribution. For instance, a shorter average light path will lead to a lower optical thickness and thereby allow higher average reaction rates at the same apparent quantum yield, *cf*. Equation (17).

## 3. Molecular Photocatalytic Reactions

**Figure 8:**
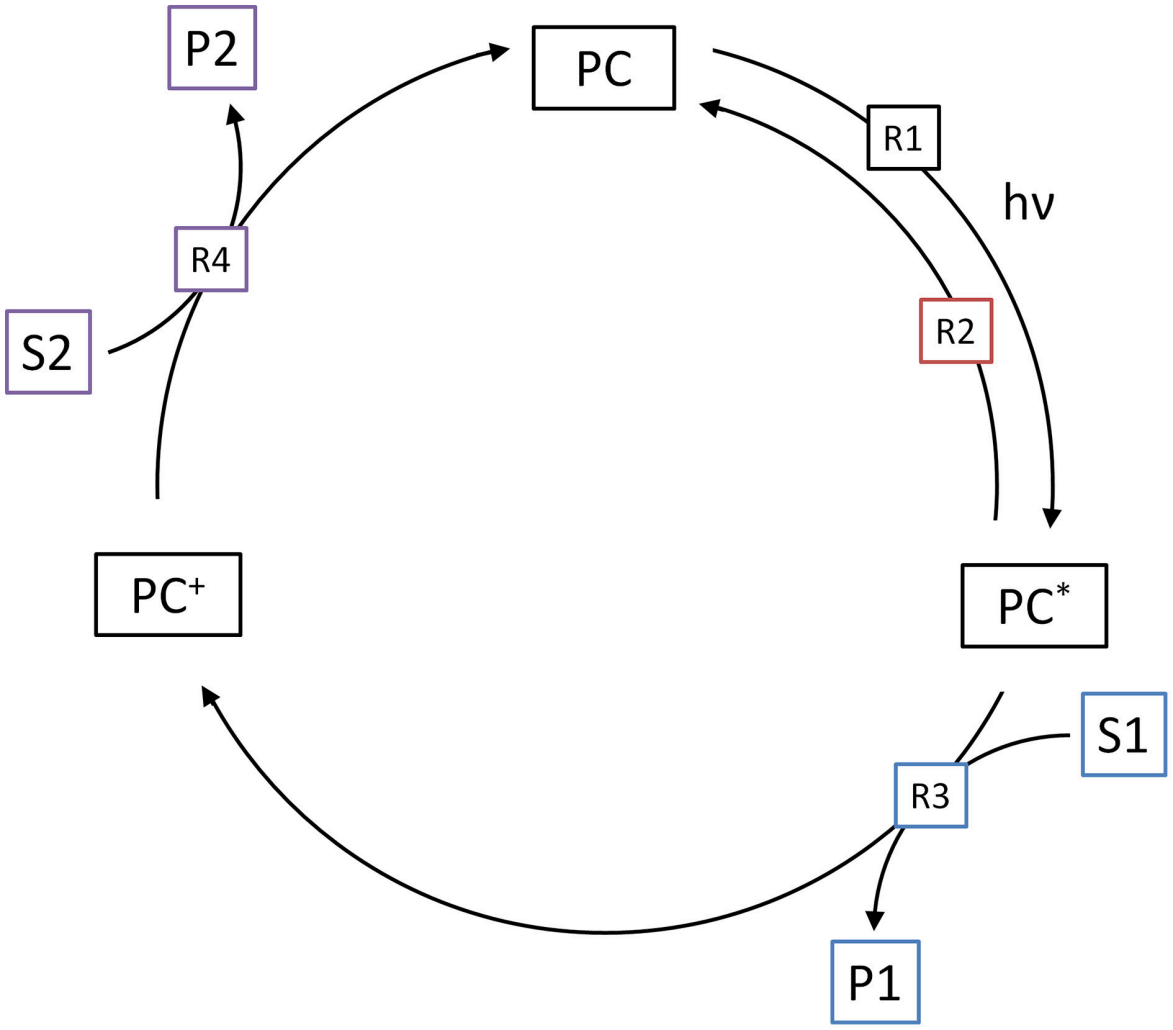
Illustration of the four elementary reactions which are the basis of the kinetic model for molecular photocatalytic reactions. Shown are the generation of the excited state (*R*1), the relaxation (*R*2), oxidative quenching (*R*3) and regeneration of the photocatalyst (*R*4).

The methodology used in the model, described above, can also be adapted to molecular (homogeneous) photocatalytic reactions. As shown in Figure 8, the first step of the catalytic cycle (*R*1) is again the excitation of the photocatalyst (*PC*) to its reactive state (PC^*^), in dependence of the local volumetric rate of photon absorption ($L_{p}^{a}$). Since the reactive state is typically the triplet state, this reaction summarizes photon absorption and inter-system crossing. The quantum yield of this reaction (ϕ) consequently represents the yield of reactive (triplet) states that are generated upon photon absorption. The reaction rate is normalized to the fraction of unexcited photocatalyst to total photocatalyst. This is done under the assumption that multi-photon absorption does not lead to additional or faster reaction events, i.e., an already excited, oxidized or reduced photocatalyst may absorb additional photons, but this does not lead to an altered reactivity. This normalization is also needed, since if otherwise, given a sufficiently high light intensity, the model could mathematically create more excited photocatalysts than total photocatalysts.

The next step of the catalytic cycle is either relaxation of the excited state (*R*2) back to the ground state or reductive/oxidative quenching (*R*3). The former is usually accompanied by fluorescence and a first-order reaction with respect to concentration of the excited state. The rate of this reaction is usually expressed as the half-life-time (τ). The competing reaction is the electron transfer to or from one of the substrates. Since from a modeling perspective, oxidative and reductive quenching are identical, here only oxidative quenching (*R*3) is considered. This is typically a second-order reaction and therefore dependent on both the concentration of the excited state photocatalyst and the target substrate (*S*_1_). The rate of this reaction is further described by the corresponding bimolecular rate constant (*k*_1_).

In the last step, the cycle needs to be closed by regenerating (reducing) the oxidized photocatalyst (*PC*^+^) back to the ground state (*R*4). This is done by reaction with the second substrate (*S*_2_), again in a second-order reaction with the corresponding bimolecular rate constant (*k*_2_). Note that either the first, second or both substrates might represent the target reaction.

(25) $$\begin{array}{l} R1\left(PC\to PC^{*}\right):\phi \cdot L_{p}^{a}\cdot \frac{\left[PC\right]}{\left[PC_{0}\right]} \end{array}$$

(26) $$\begin{array}{l} R2\left(PC^{*}\to PC\right):\frac{\left[PC^{*}\right]}{\tau } \end{array}$$

(27) $$\begin{array}{l} R3\left(PC^{*}+S_{1}\to PC^{+}+P_{1}\right):k_{1}\cdot \left[S_{1}\right]\cdot \left[PC^{*}\right] \end{array}$$

(28) $$\begin{array}{l} R4\left(PC^{+}+S_{2}\to PC+P_{2}\right):k_{2}\cdot \left[S_{2}\right]\cdot \left[PC^{+}\right] \end{array}$$

Since these reactions are usually significantly faster (ns to ms time-scale) than macroscopic mixing and changes in the substrate concentrations, it is assumed that a steady state is also present with respect to the local light intensity and substrate concentration. A steady-state approximation ([*PC*], [PC^*^], [*PC*^+^] = *const*., *R*1 = *R*2 + *R*3, *R*3 = *R*4) yields Equation (29) for [PC^*^] and Equation (30) for *R*3/4.

(29) $$\begin{array}{l} \left[PC^{*}\right]=\frac{\phi \cdot L_{p}^{a}}{\frac{\phi \cdot L_{p}^{a}}{\left[PC_{0}\right]}\left(1+\frac{k_{1}\cdot \left[S_{1}\right]}{k_{2}\cdot \left[S_{2}\right]}\right)+\frac{1}{\tau }+k_{1}\cdot \left[S_{1}\right]} \end{array}$$

(30) $$\begin{array}{l} r=R3=R4=\frac{k_{1}\cdot \left[S_{1}\right]\cdot \phi \cdot L_{p}^{a}}{\frac{\phi \cdot L_{p}^{a}}{\left[PC_{0}\right]}\left(1+\frac{k_{1}\cdot \left[S_{1}\right]}{k_{2}\cdot \left[S_{2}\right]}\right)+\frac{1}{\tau }+k_{1}\cdot \left[S_{1}\right]} \end{array}$$

This rate equation has the following limiting case which represents low light intensity. Note that contrary to heterogeneous systems, in molecular photocatalysis practically all cases fall into this category (*vide infra*).

(31) $$\begin{array}{l} L_{p}^{a}\ll \frac{\left[PC_{0}\right]\cdot \left(\frac{1}{\tau }+k_{1}\cdot \left[S_{1}\right]\right)}{1+\frac{k_{1}\cdot \left[S_{1}\right]}{k_{2}\cdot \left[S_{2}\right]}}:\langle r\rangle =\phi \cdot \langle L_{p}^{a}\rangle \cdot \frac{k_{1}\cdot \left[S_{1}\right]\cdot \tau }{1+k_{1}\cdot \left[S_{1}\right]\cdot \tau } \end{array}$$

As stated above for heterogeneous systems, in cases when the response of the reaction rate to light intensity is linear in the entire reaction medium (low light intensity), the local volumetric rate of photon absorption may be replaced by the average volumetric rate of photon absorption ($\langle L_{p}^{a}\rangle$, AVRPA), to calculate the observed average reaction rate 〈*r*〉. The AVRPA can easily be measured using actinometry or calculated from the incident photon flux and the extinction of the photocatalyst. In this linear case, the well-known Stern-Volmer equation, Equation (33), can also be derived from the relaxation rate (*R*2), Equation (32), which it is proportional to the fluorescence intensity (*F*).

(32) $$\begin{array}{l} R2=\frac{\phi \cdot \langle L_{p}^{a}\rangle }{1+k_{1}\cdot \left[S_{1}\right]\cdot \tau }\propto F \end{array}$$

(33) $$\begin{array}{l} \frac{F_{0}}{F}=1+k_{1}\cdot \left[S_{1}\right]\cdot \tau \end{array}$$

As long as the light intensity is sufficiently small and the reaction rate increases linearly with the light intensity, the reaction kinetics can easily be described by Equation (31). This represents a mixed zero- and first-order reaction similar to Langmuir-Hinshelwood or Michaelis-Menten kinetics. At a high substrate concentration, the reaction is zero-order and the reaction rate is only dependent on the light intensity and quantum yield, Equation (34).

(34) $$\begin{array}{l} \left[S_{1}\right]\cdot \tau \cdot k_{1}\gg 1:r=\phi \cdot \langle L_{p}^{a}\rangle =r_{max} \end{array}$$

At lower substrate concentration the reaction rate gradually shifts to first-order kinetics, where it is linearly dependent on the substrate concentration, quantum yield, light intensity, bimolecular rate constant and excited state life-time of the photocatalyst, Equation (35). A pseudo-inflection point between both regimes is given by the half-maximum reaction rate which is reached when [*S*_1_]·*k*_1_·τ = 1. This is exactly the behavior that is often observed if kinetics of photoredox catalysis are studied (Gazi et al., 2017; Le et al., 2017).

(35) $$\begin{array}{l} \left[S_{1}\right]\cdot \tau \cdot k_{1}\ll 1:r=\phi \cdot \langle L_{p}^{a}\rangle \cdot k_{1}\cdot \left[S_{1}\right]\cdot \tau \end{array}$$

Unfortunately, integration of this rate law (Equation 31) does not yield an explicit equation for the substrate concentration change over time, but it can readily be modeled and fitted using a numerical simulation (e.g., Euler-Cauchy method). Fitting this equation to a sufficiently detailed concentration-time-profile will also directly allow to extract both the quantum yield and bimolecular rate constant if τ and $\langle L_{p}^{a}\rangle$ are known, rendering Stern-Volmer analysis superfluous.

All of these simplifications are only applicable when the light intensity is so small, that the majority of the photocatalyst is always in its ground state. At higher light intensity, the reaction rate would yield increasingly diminishing returns with respect to the light intensity. Given the limits of linearity, it seems rather unlikely to actually reach the non-linear part in practical applications. Using the same method described above for heterogeneous systems, the maximum local absorbed light intensity present at the very beginning of the light path (*z* = 0) can again be calculated using Equation (16).

(36) $$\begin{array}{l} \epsilon \cdot ln\left(10\right)\cdot I_{0}\ll \frac{\left(\frac{1}{\tau }+k_{1}\cdot \left[S_{1}\right]\right)\cdot \left(k_{2}\cdot \left[S_{2}\right]\right)}{k_{1}\cdot \left[S_{1}\right]+k_{2}\cdot \left[S_{2}\right]} \end{array}$$

This reduces the limiting case to Equation (36). Neglecting concentrated light sources such as lasers, irradiances of up to about 10 W cm^−2^ are possible with current technology. This equals a photon flux of about 35 μmol cm^−2^ s^−1^ in the UVA to blue light region. With ϵ = 15.000 L mol^−1^ cm^−1^ and assuming the substrates are present in 10 mmol L^−1^ concentration, this means that both bimolecular rate constants need to be much larger than 1.2 × 10^5^ L mol^−1^ s^−1^, or in case of *k*_1_, the half-life time of the photocatalyst's excited state may instead be much shorter than 0.8 ms. Given that both, rate constants are typically reported in the range of 10^6^ to 10^8^ L mol^−1^ s^−1^ and the half-life time of the photocatalysts are often in the low μs regime, the limiting case seems to be fulfilled in practically all cases relevant today. However, if strongly absorbing photocatalysts are used in combination with high light intensity, slow kinetics and low substrate concentration, this limiting case needs to be revisited in order to make sure no non-linearities occur. The authors note that if a kinetic limitation takes place it is most likely caused by a slow regeneration reaction (*R*4). In those cases, similar measures defined for the heterogeneous reactions can be taken to accurately model the system and to overcome the limitations.

Similar considerations that were made for heterogeneous systems, concerning the temperature, can also be made for photoredox catalysis, however, one has to keep in mind that not just the target kinetic constant, but all the reaction constants will vary with temperature.

## 4. Conclusion

The vast majority of reports of photocatalytic reactions are one-dimensional studies that only look at the effect of one parameter. This very easily leads to misinterpretations as correlations of different parameters are invisible in this case. Moreover, the effect of some reaction parameters such as temperature are strongly masked under a variety of conditions and can therefore only be properly studied using a holistic multi-dimensional approach.

Using the average volumetric rate of photon absorption or any figure proportional to it, such as lamp power or irradiance, to analyze the kinetics of photocatalytic reactions is only a valid approach if the light intensity is so low that the reaction rate is linearly proportional to the light intensity at every point in the reaction vessel. In that case it is also possible to use pseudo Langmuir-Hinshelwood kinetics to simulate and analyze the reaction rate, as long as the substrate concentration is the only parameter varied in a set of experiments. However, in this case care need to be taken not to misinterpret the corresponding parameters (*k*′ and $K_{ads}^{\prime }$) for their actual physical meaning, as they are in fact modulated by a number of other parameters such as the light intensity.

In case of moderate to high light intensities, the abovementioned simplification does not hold true and the reaction rate instead has to be integrated over the whole reaction volume, taking the light distribution into account. However, herein we could show that for some experimental setups there exist relatively simple explicit equations to solve this rather complex problem. These can easily be fit to the experimental data to obtain the underlying physically meaningful parameters. First examples already show that this approach yields very good results and can account for the effect of light intensity, catalyst and substrate concentration as well as temperature as well as their respective inter-dependencies (Burek et al., 2019).

Temperature is mostly neglected as a parameter in photocatalytic reactions as typically no significant influence is observed. However, this is only true as long as the intrinsic reaction kinetics are not rate-limiting and the reaction is purely governed by the number of available photons. At higher light intensities, the reaction kinetics play an ever-increasing role and here, temperature effects become apparent. In fact, we could show that a classical Arrhenius approach to modulate the rate constant yields very good results here. Similarly, the effect of other parameters affecting the reaction kinetics such as substrate concentration and catalyst concentration is much more prominent at high light intensities.

From a reaction engineering point of view, it would be desirable to work at the highest technically achievable light intensity, while still maintaining high photonic efficiency. Unfortunately, the reaction rate's response to light intensity becomes increasingly non-linear with increased intensity, which led several researchers to believe that high productivity and high efficiency are contradictory. However, the non-linearity can be compensated by increasing the reaction kinetics accordingly, e.g., by using a higher temperature, substrate or catalyst concentration. Yet, there are still limits to what extent this can be done to, so the ultimate goal should be to improve the employed catalyst or to reduce the inhomogeneity of the light distribution in the reaction vessel, e.g., by using delocalized internal illumination (Burek et al., 2017).

If the aim is only to achieve a maximum apparent quantum yield, the reaction should be run at low light intensity. Under these conditions, the reaction kinetics are also much more forgiving and using lower substrate/photocatalyst concentration and temperature should not have a significant negative impact on the observed reaction rate.

Similar considerations can be applied for molecular photocatalytic reactions and the respective equations, to simulate and analyze the mixed zero- and first-order kinetics of these reactions are given herein. However, integration over the reaction volume is likely not necessary in these cases, because, due to generally better kinetics, it is unlikely that non-linearities will appear when considering the technical limits of the current non-focused light sources.

## Data Availability

All datasets generated for this study are included in the manuscript.

## Author Contributions

The author confirms being the sole contributor of this work and has approved it for publication.

### Conflict of Interest Statement

The author declares that the research was conducted in the absence of any commercial or financial relationships that could be construed as a potential conflict of interest.

## Figures and Tables

**Table:** 

***Symbol***	***Unit***	***Description***
α	-	Optical thickness
ϵ	L g-1 cm-1	Mass extinction coefficient (of heterogeneous photocatalyst)
ϵ	L mol-1 cm-1	Molar extinction coefficient (of molecular photocatalyst)
ϕ	-	Intrinsic quantum yield
θ	-	Surface coverage of the catalyst with the substrate
τ	s	Half-life time of the photocatalyst's excited state
*A*	mol g-1 s-1	Pre-exponential factor
*c*_0_	g L-1	Catalyst concentration
*c*_*R*_	mol L-1	Concentration of unexcited reactive centers
*c*_*R*, 0_	mol L-1	Concentration of total reactive centers
$c_{R}^{*}$	mol L-1	Concentration of excited reactive centers
*d*	cm	Total length of the reactor alongside the beam direction (z)
*E*_*A*_	kJ mol-1	Activation energy
$E_{A}^{*}$	kJ mol-1	Apparent activation energy
*F*	-	Fluorescence intensity
*F*_0_	-	Fluorescence intensity in the absence of a quencher
*I*_0_	mol m-2 s-1	Photon irradiance incident at the reactor surface
*I*(*z*)	mol m-2 s-1	Photon irradiance at a specific position alongside the beam direction (z)
*k*	s-1	First-order rate constant for the conversion of the substrate to the product
k^*^	mol g-1 s-1	Rate constant *k*_1_ normalized to the catalyst concentration
*k*′	mol L-1 s-1	Pseudo Langmuir-Hinshelwood rate constant
*k*_1_	L mol-1 s-1	Second-order rate constant for the conversion of the substrate to the product
*k*_2_	L mol-1 s-1	Second-order rate constant for the regeneration of the photocatalyst
*k*_*r*_	mol L-1 s-1	Recombination rate constant
*K*_*ads*_	L mol-1	Adsorption constant for the substrate on the photocatalyst
$K_{ads}^{\prime }$	L mol-1	Pseudo Langmuir-Hinshelwood adsorption constant
$L_{p}^{a}$	mol L-1 s-1	Local volumetric rate of photon absorption (LVRPA)
$L_{p}^{a}\left(z\right)$	mol L-1 s-1	LVRPA at a specific depth of reactor alongside the beam direction (z)
$L_{p,max}^{a}$	mol L-1 s-1	Maximum LVRPA at any position in the reactor
$\langle L_{p}^{a}\rangle$	mol L-1 s-1	Average volumetric rate of photon absorption (AVRPA)
[*PC*]	mol L-1	Concentration of the photocatalyst in the ground state
[*PC*_0_]	mol L-1	Total concentration of the photocatalyst in all states
[PC^*^]	mol L-1	Concentration of the photocatalyst in the excited state
[*PC*^+^]	mol L-1	Concentration of the photocatalyst in the oxidized state
*q*_*p*_	mol L-1 s-1	Volumetric photon flux density
*r*	mol L-1 s-1	Local reaction rate
*r*_*max*_	mol L-1 s-1	Maximum local reaction rate
〈*r*〉	mol L-1 s-1	Average reaction rate
*R*	J mol-1 K-1	Universal gas constant
[*S*]	mol L-1	Concentration of the substrate
[*S*_1_]	mol L-1	Concentration of substrate 1
[*S*_2_]	mol L-1	Concentration of substrate 2
*T*	K	Absolute temperature
*z*	cm	Depth into in the reactor alongside the beam direction
